# Targeted metabolomics reveals aberrant profiles of serum bile acids in patients with schizophrenia

**DOI:** 10.1038/s41537-022-00273-5

**Published:** 2022-08-18

**Authors:** Ying Qing, Pengkun Wang, Gaoping Cui, Juan Zhang, Kemei Liang, Zhong Xia, Peng Wang, Lin He, Wei Jia

**Affiliations:** 1 Bio-X Institutes, Key Laboratory for the Genetics of Developmental and Neuropsychiatric Disorders, Ministry of Education, Shanghai Jiao Tong University, Shanghai, 200030 China; 2 The Fourth People’s Hospital of Wuhu, Wuhu, 241003 China; 3 Center for Translational Medicine and Shanghai Key Laboratory of Diabetes Mellitus, Shanghai Jiao Tong University Affiliated Sixth People’s Hospital, Shanghai, 200233 China; 4 School of Chinese Medicine, Hong Kong Baptist University, Kowloon Tong, Hong Kong, 999077 China

**Keywords:** Schizophrenia, Biomarkers, Molecular neuroscience

## Abstract

Emerging evidence indicates that bile acids (BAs), which are signaling molecules that regulate metabolism and inflammation, appear to be dysregulated in schizophrenia (SZ). Further investigation is warranted to comprehensively characterize BA profiles in SZ. To address this, we analyzed serum BA profiles in 108 drug-free patients with SZ and in 108 healthy controls (HCs), divided into a discovery set (*n* = 119) and a validation set (*n* = 97), using ultraperformance liquid chromatography triple quadrupole mass spectrometry. Forty serum BAs were detected and absolutely quantified using calibration curves. Global BA profiling showed differences in SZ and HC groups in both discovery and validation sets. The concentrations of chenodeoxycholic acid, ursodeoxycholic acid, 3β-chenodeoxycholic acid, 7-ketolithocholic acid, 3-dehydrocholic acid, total BAs, and unconjugated BAs were significantly lower in patients with SZ compared with HCs in the two sample sets. The BA deconjugation potentials by gut microbiota and the affinity index of the farnesoid X receptor (FXR) were notably decreased in SZ patients compared to those of HCs. Conjugated BAs and BA deconjugation potentials differed in SZ patients with first versus recurrent episodes, although similar BA profiles were observed in both groups. In addition, a panel of 8 BA variables acted as a potential auxiliary diagnostic biomarker in discriminating SZ patients from HCs, with area under the curve values for receiver operating characteristic curves of 0.758 and 0.732 and for precision-recall curves of 0.750 and 0.714 in the discovery and validation sets, respectively. This study has provided compelling evidence of comprehensive characteristics of circulating BA metabolism in patients with SZ and promoted a deeper understanding of the role of BAs in the pathophysiology of this disease, possibly via the gut microbiota-FXR signaling pathway.

## Introduction

Schizophrenia (SZ) is a severe and complex mental disorder with a global prevalence of around 0.3%^1^, constituting a substantial health and economic burden on patients and society. Nevertheless, the etiology and pathogenesis of this disease remain elusive. Previous research demonstrates that the pathophysiology of SZ involves disturbed glucose and lipid metabolism^2^ and chronic low-level inflammation^3^. Recent evidence shows that bile acids (BAs) play a pivotal regulatory role in inflammation and glucose and lipid metabolism^4,5^ and seem to be dysregulated in SZ^6^, suggesting their potential involvement in SZ by affecting inflammatory response and metabolism. It is therefore important to comprehensively investigate BAs and their role in the development of SZ.

Bile acids are a diverse class of cholesterol-derived amphipathic metabolites that are mainly synthesized in the liver through the classic pathway and alternative pathway and metabolized by gut microbiota^7^. Notably, BAs can also be synthesized locally in the brain, although the majority of brain BAs are taken up from the systemic circulation^8^. BAs are critical signaling molecules that modulate metabolic processes by binding to nuclear and membrane receptors such as farnesoid X receptor (FXR) and Takeda G protein-coupled receptor 5 (TGR5)^9,10^, in addition to their well-established function as detergents that aid in the digestion and absorption of dietary lipids. FXR-mediated BA signaling is capable of modulating BA synthesis^11^, lipid, glucose and energy metabolism^12,13^, inflammation^14^, and also the transcription of several genes, including brain-derived neurotrophic factor (BDNF)^15^. TGR5-mediated BA signaling in immune cells has been demonstrated to decrease phagocytic activity and pro-inflammatory cytokine production, suggesting an immunomodulatory action of BAs^5,16,17^. Since extensive research has shown that aberrant glucose and lipid metabolism^18–20^, elevated levels of pro-inflammatory cytokines^3^, and abnormal expression of BDNF^21,22^ are associated with SZ, it is possible that changes in BA signaling pathways, such as alterations in BA pool size or composition, could play a role in the pathophysiology of this disease.

Bile acids and the gut microbiome mutually regulate each other in the intestines. Microbiota transform host-derived primary BAs in the gut by carrying out numerous reactions, including deconjugation via bile salt hydrolases (BSH) and 7α-dehydroxylation of the primary BAs glyco-conjugated and tauro-conjugated cholic acid (CA) and chenodeoxycholic acid (CDCA) to form their secondary BAs, deoxycholic acid (DCA) and lithocholic acid (LCA), respectively^23^. Of the gut microbiota producing BSH^4,24^, many bacteria, including *Bacteroides*, *Bifidobacterium*, *Blautia*, *Clostridium*, *Enterococcus*, *Eubacterium*, *Lactobacillus*, and *Roseburia*, exhibited alterations in patients with SZ^25^, hinting a potential aberrant deconjugation action in the disease. In turn, BAs regulate the gut microbiome by affecting microbial composition and function and leading to pathological consequences in cases of BA signaling pathway dysfunction^26,27^.

This study aims to comprehensively characterize BA profiles in the context of SZ, and to gain understanding of the role of BAs in SZ onset and development. To our knowledge, this is the most extensive study of its kind, with large sample size and a high-coverage targeted metabolomics method, allowing for thorough scrutiny of BAs. Our study demonstrates that BA pool size and composition are significantly different in patients with SZ when compared to HCs, and suggests that BAs may be involved in the pathophysiology of SZ via the gut microbiota-FXR signaling pathway.

## Results

### Demographic characteristics of the study population

This study included 216 participants, of which 108 were drug-free SZ patients and 108 were healthy controls (HCs). Fifty-nine SZ patients and 60 HCs were assigned to the discovery set, matched for age, sex, height, weight, BMI, and smoking habits. The remaining 49 SZ patients and 48 HCs were assigned to the validation set. The detailed demographic characteristics of the participants are shown in Table 1.

**Table 1:** Demographic characteristics of the study population.

Variables	Discovery Set			Validation Set		
	SZ (*N* = 59)	HC (*N* = 60)	*p*-value	SZ (*N* = 49)	HC (*N* = 48)	*p*-value
Age, y, mean (SD)^a^	37.48 (11.80)	36.80 (9.74)	0.732	36.13 (12.68)	28.29 (8.60)	<0.001
Sex (M/F)^b^	23/36	25/35	0.765	20/29	0/48	<0.001
Height, cm, mean (SD)^a^	163.49 (6.95)	165.00 (6.88)	0.241	158.20 (5.82)	160.08 (4.59)	0.09
Weight, kg, mean (SD)^a^	58.62 (7.76)	60.78 (8.72)	0.162	53.44 (8.20)	53.02 (6.14)	0.783
BMI, kg m^−2^, mean (SD)^a^	21.93 (2.46)	22.30 (2.75)	0.448	21.17 (2.53)	20.67 (2.10)	0.315
Smoker^c^, No. (%)^b^	15 (25.4)	12 (20)	0.480	0 (0)	6 (12.5)	0.011
CGI-S, median (IQR)	6 (0)	NA	NA	6 (1)	NA	NA

*M* male, *F* female, *BMI* body mass index, *CGI-S* the clinical global impression-severity of illness scale, *SD* standard deviation, *IQR* interquartile range, *NA* not applicable.

^a^The *p*-values were calculated by two-tailed Student’s *t*-test.

^b^The *p*-values were calculated by the chi-square test.

^c^Smoker represents the number of subjects with a habit of smoking.

### Abnormal serum bile acid profiles in patients with SZ

**Fig. 1:**
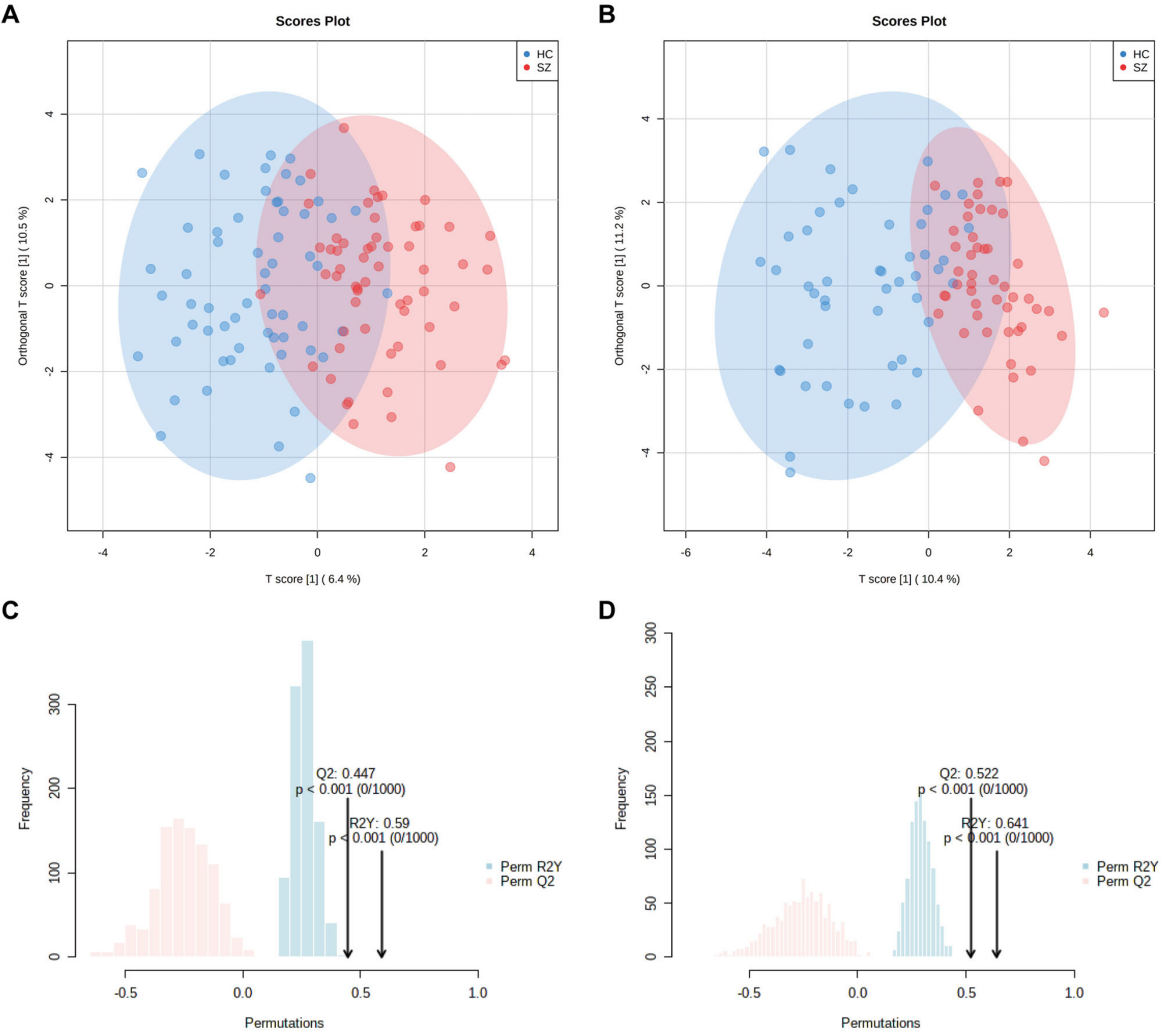
OPLS-DA based on 40 BAs shows separation between SZ and HC groups. **A** The scores plot of the discovery set with 95% confidence ellipses drawn. **B** The scores plot of the validation set with 95% confidence ellipses drawn. **C** The validation plot of 1000 permutation tests in the discovery set. **D** The validation plot of 1000 permutation tests in the validation set.

Forty BAs were detected by ultraperformance liquid chromatography triple quadrupole mass spectrometry (UPLC-TQMS), and were classified as primary BAs or secondary BAs in conjugated or unconjugated form (Table S1). To characterize serum BA profiles of patients with SZ, multivariate and univariate analyses were performed. Principal component analysis (PCA) performed on all the samples showed that all nine quality control (QC) samples were clustered closely in the PCA scores plot (Fig. S1), which demonstrated the stability of the instrument and the excellent repeatability of the analysis. Orthogonal partial least squares discriminant analysis (OPLS-DA) was performed on the discovery and validation sets to identify differences in global BA profiles between SZ and HC groups. The score plots of the models showed a separation between SZ and HC groups in both discovery and validation sets (Fig. 1A, B). Furthermore, a permutation test with 1,000 iterations was performed to assess statistical significance and avoid overfitting of the OPLS-DA model, and the result demonstrated the model’s ability to distinguish between SZ and HC groups (Fig. 1C, D). The variable importance in projection (VIP) scores of 40 BAs are listed in Table 2. Following Shapiro-Wilk tests, the Mann–Whitney *U* tests were used to compare the concentrations of 40 BAs between patients with SZ and HCs, excluding outliers less than Q1 – 1.5x IQR or greater than Q3 + 1.5x IQR. Each BA that had a VIP score > 1 and a false discovery rate (FDR)-adjusted *q*-value < 0.05 was defined as significantly different between SZ and HC groups.

**Table 2:** Median (IQR) serum concentrations (nM) of 40 BAs in SZ and HC groups in the discovery set.

BAs	SZ (*n* = 59)	HC (*n* = 60)	ratio^a^	*p*-value^b^	*q*-value^c^	VIP^d^
**CA**	12.6 (25.38)	23.3 (36.19)	0.54	0.009	**0.04**	**1.68**
GCA	31.14 (32.63)	45.2 (82.92)	0.69	0.031	0.09	1.18
TCA	3.13 (3.56)	3.23 (6.11)	0.97	0.565	0.752	0.72
**CDCA**	31.89 (89.3)	105.58 (114.83)	0.30	0.001	**0.01**	**1.64**
GCDCA	269.41 (372.09)	278.62 (328.47)	0.97	0.635	0.752	0.04
TCDCA	18.87 (28.86)	20.62 (29.87)	0.92	0.656	0.752	0.16
**DCA**	47.22 (48.23)	78.19 (87.56)	0.60	0.001	**0.01**	**1.62**
GDCA	46.15 (64.18)	61.4 (83.76)	0.75	0.087	0.204	0.64
TDCA	4.19 (6.85)	4.63 (5.33)	0.90	0.539	0.752	0.03
**UDCA**	9.35 (23.51)	24.15 (28.43)	0.39	0.001	**0.01**	**1.61**
GUDCA	36.49 (55.14)	39.96 (37.51)	0.91	1	1	0.73
TUDCA	1.01 (1.62)	1.06 (1.39)	0.95	0.956	1	0.39
LCA	1.69 (3.18)	2.68 (3.05)	0.63	0.267	0.428	0.04
GLCA	2.67 (6.47)	3.07 (6.02)	0.87	0.658	0.752	0.03
TLCA	0.16 (0.56)	0.16 (0.62)	1.00	0.634	0.752	0.4
HCA	2.48 (4.64)	3.52 (3.89)	0.70	0.213	0.398	0.29
GHCA	3.01 (2.84)	3.85 (4.04)	0.78	0.031	0.09	1.04
THCA	1.66 (1.34)	2 (0.7)	0.83	0.006	0.04	0.75
βUDCA	41.72 (56.49)	50.7 (42.93)	0.82	0.08	0.2	0.03
**βCDCA**	19.73 (24.7)	30.97 (25.19)	0.64	0.001	**0.01**	**1.97**
**βDCA**	10.33 (16.91)	18.2 (25.93)	0.57	0.007	**0.04**	**1.31**
βUCA	0.23 (0.57)	0.26 (0.43)	0.88	0.683	0.759	0.07
βCA	1.02 (0.81)	1.25 (0.98)	0.82	0.205	0.398	0.75
βMCA	0.26 (0.75)	0.29 (0.63)	0.90	0.613	0.752	0.3
UCA	0.21 (1.23)	0.26 (0.5)	0.81	0.354	0.544	1.29
TαMCA	0.93 (0.51)	1.11 (1.31)	0.84	0.018	0.06	1.33
alloLCA	0.28 (0.39)	0.21 (0.27)	1.33	0.231	0.398	1.11
isoLCA	2.19 (4.73)	3.4 (7.2)	0.64	0.073	0.194	0.79
NorDCA	0.57 (0.99)	0.53 (0.61)	1.08	0.232	0.398	0.84
6-ketoLCA	0.72 (0.47)	0.77 (0.42)	0.94	0.165	0.348	0.79
**7-ketoLCA**	2.02 (3.23)	3.85 (3.73)	0.52	0.013	**0.047**	**1.27**
12-ketoLCA	1.66 (2.74)	2.18 (4.06)	0.76	0.239	0.398	0.17
**apoCA**	1.44 (2.41)	0.9 (1.22)	1.60	0.012	**0.047**	**1.94**
LCA-3S	1.82 (5.84)	1.74 (5.03)	1.05	0.978	1	0.25
GLCA-3S	47.13 (75.5)	41.36 (58.9)	1.14	0.656	0.752	0.09
HDCA	0.85 (0.56)	0.93 (0.68)	0.91	0.123	0.273	0.52
**NorCA**	2.28 (2.34)	1.47 (1.17)	1.55	0.003	**0.027**	**1.79**
**3-DHCA**	0.2 (0.31)	0.32 (0.32)	0.63	0.009	**0.04**	**1.28**
7-ketoDCA	0.71 (1.18)	0.8 (0.94)	0.89	0.719	0.778	0.78
CDCA-3Gln	5.43 (5.6)	5.28 (7.25)	1.03	0.561	0.752	0.51

*BAs* bile acids, *VIP* variable importance in the projection, *CA* cholic acid, *GCA* glycocholic acid, *TCA* taurocholic acid, *CDCA* chenodeoxycholic acid, *GCDCA* glycochenodeoxycholic acid, *TCDCA* taurochenodeoxycholic acid, *DCA* deoxycholic acid, *GDCA* glycodeoxycholic acid, *TDCA* taurodeoxycholic acid, *UDCA* ursodeoxycholic acid, *GUDCA* glycoursodeoxycholic acid, TUDCA tauroursodeoxycholic acid, *LCA* lithocholic acid, *GLCA* glycolithocholic acid, *TLCA* taurolithocholic acid, *HCA* hyocholic acid, *GHCA* glycohyocholic acid, *THCA* taurohyocholic acid, βUDCA 3β-ursodeoxycholic acid, *βCDCA* 3β-chenodeoxycholic acid, *βDCA* 3β-deoxycholic acid, *βUCA* β-ursocholic acid, *βCA* 3β-cholic acid, *βMCA* β-muricholic acid, *UCA* ursocholic acid, *TαMCA* tauro α-muricholic acid, alloLCA allolithocholic acid, *isoLCA* isolithocholic acid, *NorDCA* 23-nordeoxycholic acid, *6-ketoLCA* 6-ketolithocholic acid, *7-ketoLCA* 7-ketolithocholic acid, *12-ketoLCA* 12-ketolithocholic acid, *apoCA* apocholic acid, *LCA-3S* lithocholic acid-3-sulfate, *GLCA-3S* glycolithocholic acid-3-sulfate, *HDCA* α-hyodeoxycholic acid, *NorCA* norcholic acid, *3-DHCA* 3-dehydrocholic acid, *7-ketoDCA* 7-ketodeoxycholic acid, *CDCA-3Gln* chenodeoxycholic acid-3-β-d-glucuronide.

^a^Ratios were calculated from the intra-group medians of BAs between SZ and HC groups.

^b^*P*-values were calculated by Mann−Whitney *U* tests.

^c^FDR adjusted *q*-values were calculated based on *p*-values estimated by Mann−Whitney *U* tests.

^d^ VIP scores were obtained from OPLS-DA. The bold BAs indicate significantly differential BAs between SZ and HC groups.

**Fig. 2:**
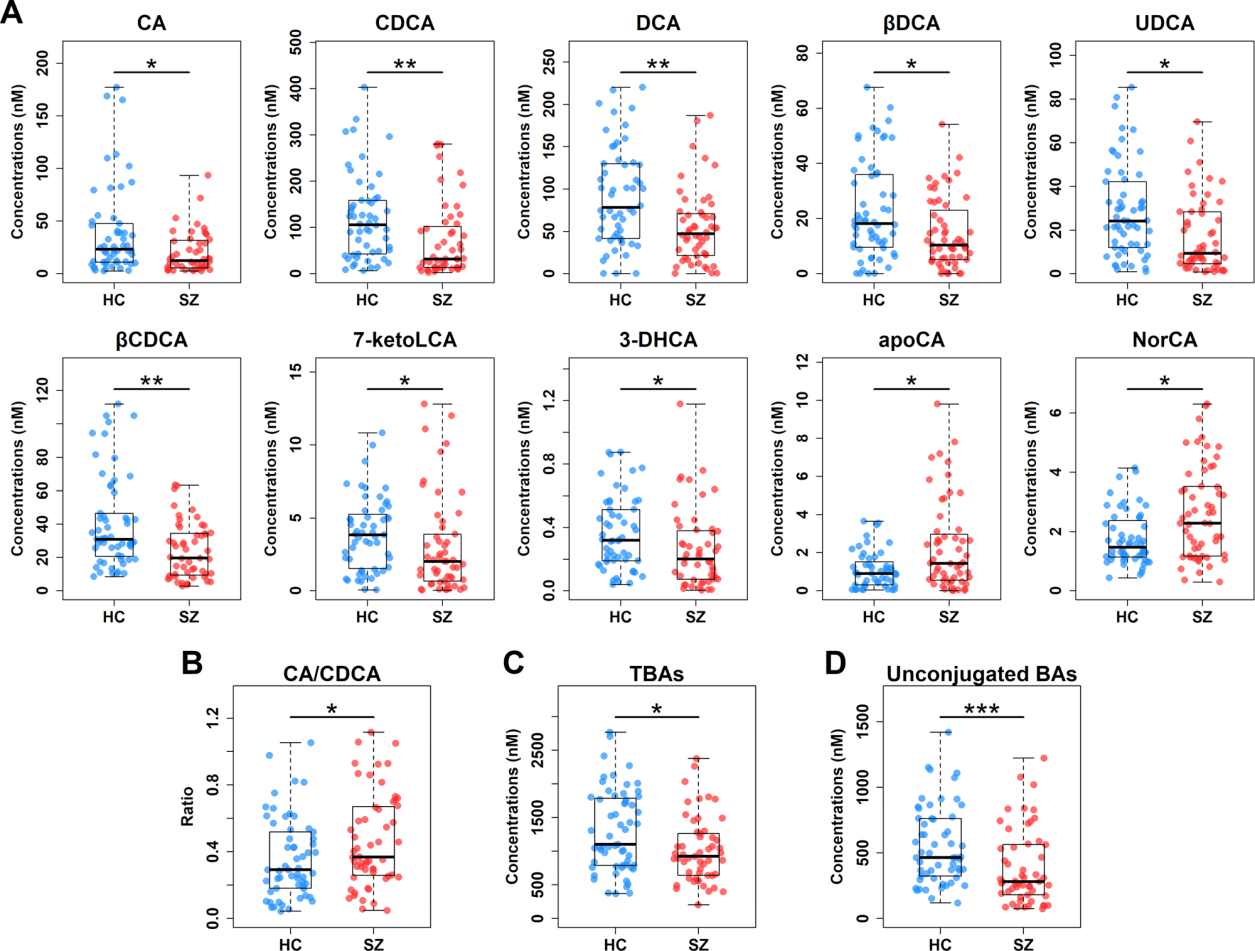
Serum BA profile is significantly altered in SZ. **A** Scatter box plots for 10 significantly differential BAs in the discovery set. The comparisons between the two groups were conducted by the Mann–Whitney *U* tests, excluding outliers and correcting with FDR. *q < 0.05; **q < 0.01. Scatter box plots for the ratio of CA to CDCA (**B**), total BAs (TBAs) (**C**), and total unconjugated BAs (**D**). The comparisons between the two groups were conducted by the Mann–Whitney *U* tests, excluding outliers. *p < 0.05; ***p < 0.001. Center lines of box plots show median values, box hinges indicate 1st and 3rd quartiles, and whisker represent the furthest data points within 1.5 interquartile ranges of the hinges.

As shown in Table 2 and Fig. 2A, the concentrations of 10 BAs were significantly different between SZ patients and HCs in the discovery set. CA and CDCA, two primary bile acids synthesized from cholesterol, were notably decreased in sera of patients with SZ when compared to HCs. DCA, the bacterial 7α-dehydroxylation product of CA, was also significantly reduced in SZ patients relative to HCs. Similarly, DCA’s epimer 3β-deoxycholic acid (βDCA) was decreased in SZ patients compared to HCs. In addition, patients with SZ had significant lower levels of ursodeoxycholic acid (UDCA) and 3β-chenodeoxycholic acid (βCDCA), which are two epimers of CDCA, in comparison with those of HCs. Moreover, SZ patients had notably reduced levels of 7-ketolithocholic acid (7-ketoLCA) and 3-dehydrocholic acid (3-DHCA) and increased levels of apocholic acid (apoCA) and norcholic acid (NorCA) when compared to those of HCs. Interestingly, as the decrease in CDCA was more profound than that observed in CA, a notable increase in the ratio of CA to CDCA was observed in patients with SZ compared to that of HCs (Fig. 2B), implying that the alternative BA synthetic pathway is impaired in SZ.

Furthermore, patients with SZ exhibited notable decline in the concentrations of total BAs (Fig. 2C) and unconjugated BAs (Fig. 2D) compared with HCs. In contrast, the total conjugated BAs were comparable with no significant differences between the two groups (Fig. S2A). Likewise, total primary and secondary BAs were also comparable between SZ patients and HCs (Figs. S2B and S2C). These findings were suggestive of weakened deconjugation by gut microbiota in SZ. In the independent validation set, the above 10 individual BAs, total BAs, and unconjugated BAs consistently exhibited similar changing trends in patients with SZ, among which CDCA, UDCA, βCDCA, 7-ketoLCA, 3-DHCA, total BAs, and unconjugated BAs reached the threshold of statistical significance (Table S2 and Fig. S3). The ratio of CA to CDCA was marginally significant between the two groups (*p* = 0.073, ratio = 1.56).

### Depleted bile acid deconjugation potentials and FXR affinity index in schizophrenic patients

**Fig. 3:**
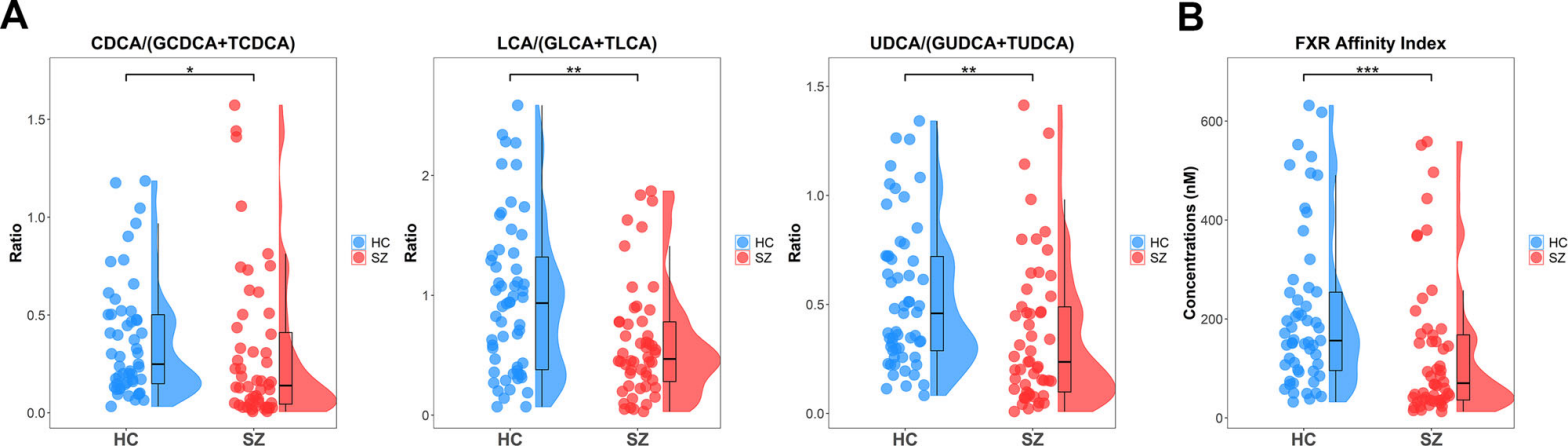
Bile acid deconjugation potentials and FXR affinity index are lower in SZ than in HCs. Raincloud plots (jittered raw data, boxplots, and probability distribution of the data) for deconjugation potentials of CDCA, LCA and UDCA (**A**), and the FXR affinity index (**B**). The comparisons between the two groups were conducted by the Mann–Whitney *U* tests, excluding outliers. *p < 0.05; **p < 0.01; ***p < 0.001.

To further evaluate BA deconjugation potentials, we calculated the pairwise ratios of unconjugated products to their conjugated precursors. These ratios were CA/(GCA and TCA), CDCA/(GCDCA and TCDCA), DCA/(GDCA and TDCA), UDCA/(GUDCA and TUDCA), LCA/(GLCA and TLCA), and HCA/(GHCA and THCA). Compared to HCs, patients with SZ had significantly lower ratios for unconjugated to conjugated CDCA and its derivatives LCA and UDCA (Fig. 3A), suggesting a hampered CDCA species-specific bacterial deconjugation manner in SZ. Additionally, FXR affinity indices were calculated and compared between the two groups, given that induction of FXR target gene expression has been shown to repress the expression of enzymes involved in hepatic BA synthesis. Patients with SZ had lower FXR affinity indices than HCs (Fig. 3B), implying amelioration of FXR-mediated repression of CYP7A1 to produce BAs via the classic pathway.

### Altered conjugated bile acids and bile acid deconjugation potentials in patients with first versus recurrent episodes of SZ

**Fig. 4:**
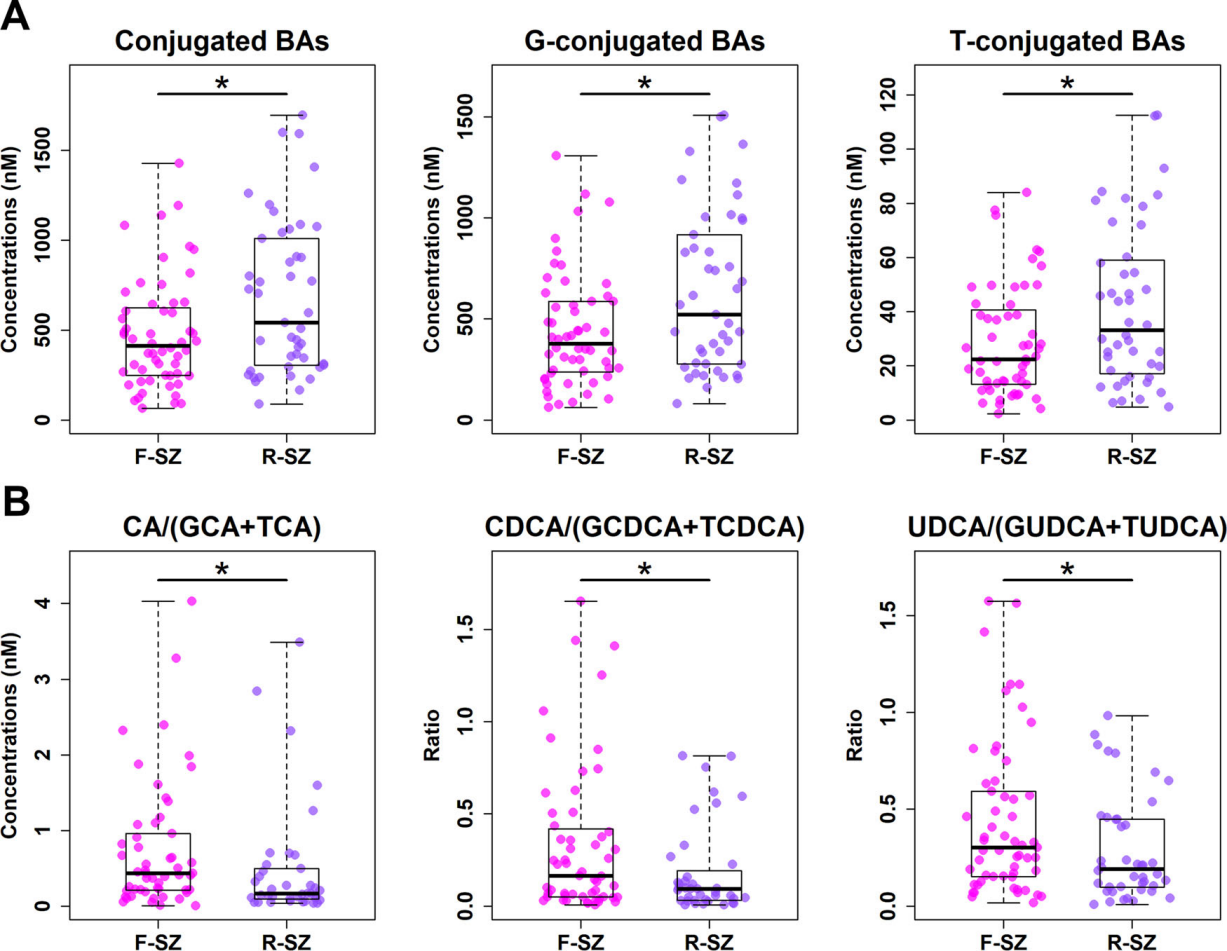
Conjugated BAs and BA deconjugation potentials differ in SZ patients with first (F-SZ) versus recurrent episodes (R-SZ). Scatter box plots for total conjugated BAs, glycine conjugated BAs and taurine conjugated BAs (**A**), and deconjugation potentials of CA, CDCA, and UDCA (**B**). The comparisons between the two groups were conducted by the Mann–Whitney *U* tests, excluding outliers. *p < 0.05.

Among the 108 patients with SZ included in the study, 62 patients were diagnosed with a first episode (F-SZ) and 46 with recurrent episodes (R-SZ). To evaluate serum BA profiles between these two groups, we compared concentrations of 40 BAs, excluding outliers, and found that 39 BAs had *p*-values > 0.05 using the Mann–Whitney *U* test, with only apoCA (*p* = 0.007; *q* = 0.298) being screened out after FDR correction with a *q*-value > 0.05. These results showed that BA profiles were similar between F-SZ and R-SZ groups. Interestingly, R-SZ had significantly higher levels of total conjugated BAs (both glyco-conjugated and tauro-conjugated BAs) than F-SZ (Fig. 4A), but showed no remarkable changes in either total BAs or unconjugated BAs. In addition, the ratios of unconjugated to conjugated CA, CDCA, and UDCA species were significantly lower in the R-SZ group than in the F-SZ group (Fig. 4B). These data hinted that recurring SZ states might affect BA conjugation and deconjugation.

### Bile acids as candidate auxiliary diagnostic markers for SZ

**Fig. 5:**
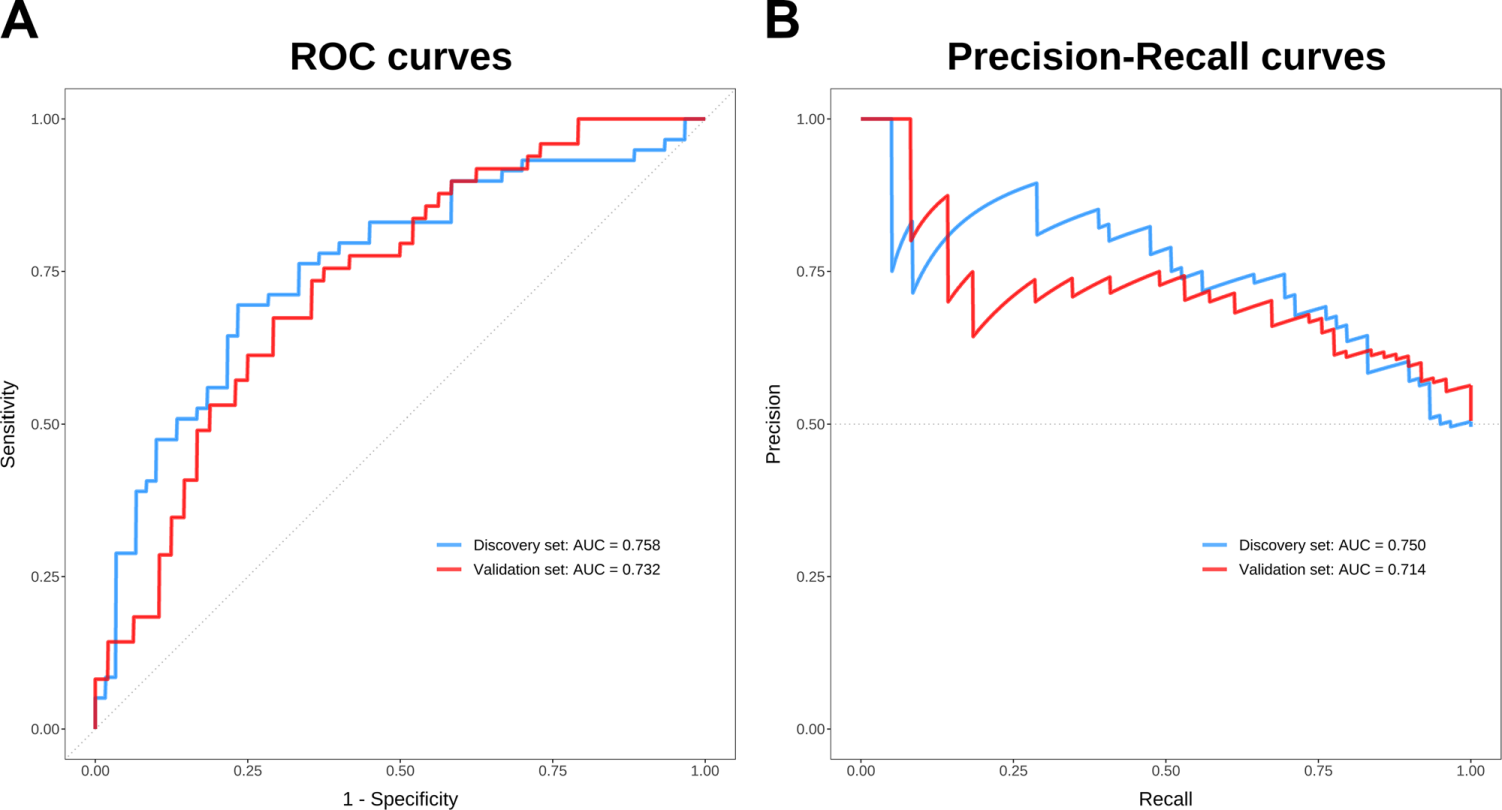
Receiver operating characteristic (ROC) and precision-recall (P-R) curves for the logistic regression models. **A** The AUC values for ROC curves distinguishing SZ from HCs in the discovery and validation sets were 0.758 and 0.732, respectively. **B** The AUC values for P-R curves distinguishing SZ from HCs in the discovery and validation sets were 0.750 and 0.714, respectively.

To explore the diagnostic effectiveness of BAs for SZ prediction, receiver operating characteristic (ROC) curves, precision-recall (P-R) curves, and their area under the curves (AUC) for 40 individually detected BAs and 5 summed BAs, including total BAs, total primary BAs, total secondary BAs, total conjugated BAs, and total unconjugated BAs, were calculated to evaluate the performance of the logistic regression models in the discovery set. Further, different biomarker panels based on the above 45 BA variables were screened to optimize the diagnostic model. According to the posterior probability that each variable is non-zero for Bayesian model averaging, a combination of 8 BA variables, including TCDCA, βCDCA, 6-ketoLCA, apoCA, NorCA, GHCA, THCA, and total primary BAs, was identified to discriminate SZ patients from HCs. When using their respectively best cut-off values in the two sample sets, AUC values for ROC curves were 0.758 (sensitivity: 0.695; specificity: 0.767) and 0.732 (sensitivity: 0.673; specificity: 0.708) (Fig. 5A), and for P-R curves were 0.750 (precision: 0.745; recall: 0.695) and 0.714 (precision: 0.702; recall: 0.673) (Fig. 5B) in the discovery and validation sets, respectively. And F1 scores for the two sample sets were 0.719 and 0.687, respectively. When using the best cut-off value of the discovery set to predict for the validation set, AUC values for ROC and P-R curves were 0.671 (sensitivity: 0.612; specificity: 0.729) and 0.650 (precision: 0.698; recall: 0.612), and F1 score was 0.652 in the validation set. Since BA profiles of F-SZ and R-SZ patients were similar, they could not be used to differentiate these two groups.

## Discussion

Emerging evidence indicates that BAs are present in the brain and are associated with brain diseases, such as schizophrenia^6^, Alzheimer’s disease^28,29^, and autism^30^, highlighting the linkage between BAs and mental health. Nevertheless, data on the BA profiles in patients with SZ are scarce, which has limited our understanding of the role of BAs in the disease. The current study provides new evidence for the comprehensive characterization of circulating BA profiles in patients with SZ and increases our understanding of BA profiles and their potential role in this disease.

Circulating BAs have the ability to cross the blood-brain barrier (BBB) via either passive diffusion or active transport, and BAs and their receptors have already been identified in the human brain, alluding to BA-mediated signaling from the peripheral circulation to the central nervous system (CNS)^31^. CA and CDCA are major primary BAs synthesized from cholesterol in the liver, via the classical and alternative pathways, respectively. Since CA and CDCA can diffuse across phospholipid bilayers^32^, they may diffuse across the BBB, and their levels in the brain are reported to correlate with those in the serum^33^. As CDCA is a potent antagonist at N-methyl-D-aspartate (NMDA) and γ-aminobutyric acid (GABA) receptors^34^, our finding of significant reduction of CDCA levels in patients with SZ relative to HCs suggests aberrant glutamate and GABA neurotransmitter systems in SZ. CDCA generation from cholesterol was controlled by CYP27A1 via the acidic (alternative) pathway^7^. More than 50 mutations in the gene *CYP27A1* have been identified associated with cerebrotendinous xanthomatosis (CTX)^35^, a rare autosomal recessive disease of BA synthesis that presents possible psychiatric manifestations^36^. Treatment with CDCA has been shown to improve the psychiatric symptoms of patients with CTX^37^. As a result of a greater decrease in CDCA levels than in CA levels, we found a higher ratio of CA to CDCA in patients with SZ. Sterol 12α-hydroxylase (CYP8B1) catalyzes CA synthesis and thus determines the ratio of CA to CDCA^38^, and it can be inhibited by CDCA and pro-inflammatory cytokine interleukin-1β (IL-1β)^39,40^. In addition, our results showed that UDCA and its precursor 7-keto LCA were depleted in SZ patients. UDCA is widely used for treatment of cholestatic liver disease^41^, and a recent case report indicates that UDCA supplementation shows clinical efficacy and safety on treatment-refractory SZ^42^, suggesting that UDCA might be a viable therapeutic target for personalized SZ treatment. Similar to our finding of a reduction in 3-DHCA (also known as 3-oxocholic acid) in patients with SZ, decreased levels of 3-DHCA have been observed in lung cancer patients with cachexia, and were positively correlated with the gut species *Lactobacillus gasseri*^43^, suggesting a potential role of gut microbiota in altering circulating BA levels.

In addition to changes in BA composition, we also observed a marked decline in BA pool size in patients with SZ. A previous genetic study identified 3 missense variants of the gene *AMACR* associated with SZ^44^, which encodes 2-methylacyl-CoA racemase and is involved in the multi-step reactions of BA synthesis, suggesting possible defects in BA synthesis in SZ. In addition, a recent study identified fibroblast growth factor 21 (FGF21), the most closely related subfamily member of FGF19, as a negative regulator of BA synthesis^45^, while our previous study has already demonstrated a marked increase in serum FGF21 levels in patients with SZ^46^, suggesting the depletion in BA production in SZ. Our observation of a notable decrease in total BA concentrations in patients with SZ is thus likely a result of BA synthesis malfunction. Decreased concentrations of total BAs have been reported in patients with type 2 diabetic peripheral neuropathy and patients with coronary artery disease^47,48^, while increased total BAs have been found in patients with type 2 diabetes and in patients with nonalcoholic steatohepatitis^4,49^.

Gut microbiota facilitate the deconjugation of conjugated BAs to form unconjugated BAs, followed by epimerization and dehydroxylation to produce secondary BAs^50^. We identified a significant depletion in unconjugated BA pool size in patients with SZ, suggesting impaired deconjugation by gut bacteria in the disease. We also found reduced deconjugation potentials in patients with SZ, further supporting the notion that deconjugation of BAs by gut microbiota is weakened in SZ. Interestingly, BSHs responsible for BA deconjugation in different bacterial phylotypes exhibit distinct deconjugation selectivity on substrates. For example, BSH-T2 (*Streptococcus, Enterococcus, Lactobacillus, Liseria, Methanosphaera*) groups show the highest specific activity with GCDCA, while the T1 (*Eubacterium*, *Blautia*, *Clostridium*, *Roseburia*, *Ruminococcus*), T3 (*Lactobacillus*), and T4 (*Bifidobacterium* and *Collinsella*) groups display the highest specific activities with GCA^51^. Recent studies have reported decreased abundances of gut *Streptococcus*, *Enterococcus*, *Blautia*, and *Roseburia* in patients with SZ^52,53^. These findings provide clues for interpretation of our findings of decreased CDCA species-specific deconjugation potentials in SZ. Furthermore, our results of significantly lower BA deconjugation potentials in R-SZ patients than in F-SZ patients suggest that BA deconjugation by gut microbiota is likely involved in schizophrenic relapse. We therefore conjecture that dysbiotic bacteria will cause a weak deconjugative capacity for specific BAs, resulting in the depletion of unconjugated BAs, and might be further implicated in the onset risk of SZ.

The disturbed availability of specific BAs such as CDCA influences FXR signaling, which plays a crucial role in the modulation of BA homeostasis and lipid and glucose metabolism^54^. Activated FXR inhibits BA production through a feedback mechanism by repressing CYP7A1 via small heterodimer partner (SHP) induction^55^. Our report of low FXR affinity indices is indicative of blunted activation of FXR, which would in turn enhance hepatic BA synthesis in SZ. In addition, FXR activation also decreases free fatty acids (FFAs) and triglycerides (TGs) by suppressing de novo fatty acid synthesis^56,57^ and promoting hepatic fatty acid oxidation^58^. Patients with SZ have been shown to have elevated levels of FFAs^59^ and TGs^60^, which is in accordance with our finding of blunted activation of FXR in the disease. In addition, FXR activation facilitates the inhibition of inflammation and maintenance of intestinal epithelial barrier in inflammatory bowel disease^61^ and promotes the suppression of C-reactive protein (CRP) expression by interleukin-6 (IL-6)^62^. Our results showing decreased activation of FXR signaling may contribute to an increase in intestinal permeability^63,64^ and upregulation of CRP^65^ and IL-6^66^ in patients with SZ.

The current study provides a comprehensive snapshot of serum BA profiles in patients with SZ and identifies changes in BA pool size and composition, providing new insights into the pathophysiology of SZ. The strength of this study lies in its large sample size, independent sample sets, and its high-coverage targeted metabolomics approach. Nevertheless, this study still has some limitations. First, it only demonstrates that SZ is associated with abnormal BA profiles, but cannot prove the causal relationship between BAs and SZ. Second, it lacks metagenomic data to determine the microbial enzymes catalyzing deconjugation reaction. In the future, preclinical or clinical studies on the microbiota-BA-FXR signaling pathway are warranted to determine the causality between BAs and SZ.

## Methods

### Study design

We recruited a total of 216 participants in Anhui Province, China, of which 108 patients were diagnosed with SZ according to the criteria of the Diagnostic and Statistical Manual of Mental Disorders, Fourth Edition (DSM-IV), and the remaining 108 subjects were HCs. Of the 108 patients, 62 were experiencing a first episode of psychosis, and 46 were hospitalized for relapse after at least 1 month without any antipsychotics. Patients who met the criteria for any other axis I disorder or who received any form of mood-stabilizing drugs in the recent 2 weeks before blood sample collection were excluded from the study. In both SZ and HC groups, participants with metabolic disorders (such as type I or type II diabetes) and/or with heavy consumption of alcohol were also excluded from this study. All patients with SZ were antipsychotics free and all participants were free of substance abuse, suicidal ideation, and unstable medical illness. The enrolled participants were assigned into the discovery data set (59 SZ and 60 HC) or the validation data set (49 SZ and 48 HC), consistent with our previous studies^20,59^. This study was approved by the local ethics committee of the Fourth People’s Hospital of Wuhu and was carried out in accordance with the Declaration of Helsinki. All participants signed written informed consent forms before undergoing any procedures.

### Serum sample collection

Overnight fasting whole blood samples were collected from all subjects and were stored at room temperature, which clotted naturally after approximately 1 h. Serum samples were then obtained after centrifugation at 10,000 rpm for 10 min. All samples were immediately aliquoted and stored at −80 °C until further analysis.

### Quantification of bile acids

Reference standards of 61 BAs were purchased from Steraloids Inc. (Newport, RI, USA) and TRC Chemicals (Toronto, ON, Canada). Ten stable isotope-labeled standards (CA-D4, UDCA-D4, DCA-D4, LCA-D4, βCA-D5, GCA-D4, TCA-D4, GCDCA-D4, TCDCA-D9, and GDCA-D4,) were obtained from C/D/N Isotopes Inc. (Quebec, Canada) and Steraloids Inc. (Newport, RI, USA). Methanol (Optima LC-MS), acetonitrile (Optima LC-MS), isopropanol (Optima LC-MS), and formic acid (Optima LC-MS) were obtained from Thermo-Fisher Scientific (Fairlawn, NJ, USA). Ultrapure water was produced with a Milli-Q Reference system equipped with an LC-MS Pak filter (Millipore, Billerica, MA, USA). The standards and stable isotope-labeled standards were accurately weighed and prepared in methanol to obtain individual stock solutions at 5.0 mM concentration. Depending on the solubility of the standard, either water or methanol was used to prepare the stock solution. All stock solutions were stored at −80 °C. The individual BA stock solutions were mixed and prepared in a BA-free serum matrix to obtain a series of BA standards at final concentrations of 2500, 500, 250, 50, 10, 2.5, and 1 nM. Quality control samples were prepared at concentrations of 1500, 150, and 5 nM. Internal standard (IS) concentrations were kept constant at all calibration points (150 nM for 10 stable isotope-labeled standards).

A Waters ACQUITY UPLC system equipped with a binary solvent delivery manager and sample manager (Waters, Milford, MA) was used in this study. The mass spectrometer was a Waters XEVO TQ-S instrument with an ESI source (Waters). The LC-MS system was controlled using the MassLynx 4.1 software. The chromatographic separations were performed using an ACQUITY UPLC CORTECS C18 1.6 μM VanGuard pre-column (2.1 × 5 mm) and ACQUITY UPLC CORTECS C18 1.6 μM analytical column (2.1 × 100 mm).

A 20-μL aliquot of serum sample was added to 180 μL of acetonitrile/methanol (8:2) containing 10 internal standards, and the mixtures were vortexed for 2 min and allowed to stand for 10 min before being centrifuged at 20,000 *g* for 10 min at 4 °C. A 160-μL aliquot of the supernatant was transferred to a clean tube and vacuum-dried. The residue was redissolved with equal amounts of acetonitrile/methanol (80/20, v/v) and ultrapure water (0.1% formic acid) to a final volume of 40 μL, and was then centrifuged at 13,500 *g* for 20 min at 4 °C. After centrifugation, the supernatant was used for UPLC–MS/MS analysis. The injection volume of all samples was 5 μL. The mobile phases were water with formic acid (pH = 3.25) (A) and acetonitrile/methanol (v/v = 80/20) (B), with a flow rate of 0.4 mL/min. The elution gradient conditions were as follows: 0-1 min (5% B), 1–3 min (5–30% B), 3–8 min (30–40% B), 8–15 min (40–100% B), 15–16 min (100–5% B), and 16–17 min (5% B). The mass spectrometer was operated in negative ion mode with the following optimal conditions: capillary voltage of 2 kV, source temperature of 120 °C, desolvation temperature of 550 °C, and desolvation gas flow rate of 1000 L/h.

To diminish analytical bias within the entire analytical process, QC samples (pooled biological samples), calibrators, and blank samples were analyzed across the entire sample set and the serum samples from SZ and HC groups were alternately injected. The calibrators consisted of a blank sample (without IS), zero sample (with IS), and series of 7 concentrations covering the expected range for BAs present in the samples. The calibration curve and the corresponding regression coefficients were obtained using IS adjustment. QC samples and IS were used for quality control of BA analysis. The UPLC-TQMS raw data files were processed and quantified using the TargetLynx Applications Manager (version 4.1, Waters). Finally, using 61 BA standards to identify and quantify the BA concentrations, 40 BAs were identified and quantified in real samples.

### Calculation of bile acid deconjugation potentials and FXR affinity index

According to the deconjugation process of BAs, BA deconjugation potentials were analyzed using 6 ratios of unconjugated to conjugated BAs, including CA/(GCA and TCA), CDCA/(GCDCA and TCDCA), DCA/(GDCA and TDCA), UDCA/(GUDCA and TUDCA), LCA/(GLCA and TLCA), and HCA/(GHCA and THCA). As in a previous study^67^, an FXR affinity index was calculated based on the following formula: FXR affinity index = CA (nM) × 0.81 + CDCA × 1 + DCA × 0.4 + LCA × 0.04.

### Statistical analysis

For multivariate analysis, PCA and OPLS-DA were conducted using MetaboAnalyst version 5.0^68^ and obtained the corresponding VIP value for each BA. For univariate analysis, the Shapiro−Wilk test was first used to examine the distribution of continuous variables. A Student’s *t-*test was then carried out to investigate differences in normally distributed variables between groups. The Mann−Whitney *U* test was performed to investigate differences in BA measurements between groups, excluding outliers less than Q1 – 1.5 x IQR or greater than Q3 + 1.5 x IQR. The resultant *p* values for BAs were subsequently adjusted to account for multiple testing by the false discovery rate (FDR) method. Logistic regression models were constructed based on the BA concentrations, and AUCs of ROC and P-R curves as well as F1 scores were calculated to evaluate the performance of the fitted logistic regression models. A *p-*value less than 0.05 was considered statistically significant. Both of the discovery and validation sets followed the above analytical procedures. All analyses were carried out using SPSS 24 and R 4.1.0.

## Supplementary information

Supplementary material

## Data Availability

The mass spectrometry raw data associated with this study have been deposited on the open metabolomics database MetaboLights (ID: MTBLS5535).

## Code availability

The R code that supports the findings of this study are available from the corresponding authors upon reasonable request.
