# Supplementary material for: Targeted metabolomics reveals aberrant profiles of serum bile acids in patients with schizophrenia
Source: Schizophrenia (Heidelb). 2022 Aug 18;8(1):65. doi: 10.1038/s41537-022-00273-5 (PMC9388515; doi:10.1038/s41537-022-00273-5)
Supplement: Supplementary file 1 — Supplementary material [file 41537_2022_273_MOESM1_ESM.docx]

**Targeted metabolomics reveals aberrant profiles of serum bile acids in patients with schizophrenia**

Ying Qing^1#*^, Pengkun Wang^1#^, Gaoping Cui^1^, Juan Zhang^1^, Kemei Liang^2^, Zhong Xia^2^, Peng Wang^2^, Lin He^1^, Wei Jia^3,4*^.

^#^ These authors contributed equally to this work.

* Corresponding authors: Ying Qing and Wei Jia.

**Author affiliations**

^1^ Bio-X Institutes, Key Laboratory for the Genetics of Developmental and Neuropsychiatric Disorders, Ministry of Education, Shanghai Jiao Tong University, Shanghai 200030, China.

^2^ The Fourth People’s Hospital of Wuhu, Wuhu 241003, China.

^3^ Center for Translational Medicine and Shanghai Key Laboratory of Diabetes Mellitus, Shanghai Jiao Tong University Affiliated Sixth People's Hospital, Shanghai 200233, China.

^4^ School of Chinese Medicine, Hong Kong Baptist University, Kowloon Tong, Hong Kong 999077, China.

Table S1: Abbreviations and taxonomic relations of bile acid variables.

Table S2: Median (IQR) serum concentrations (nM) of 40 BAs in SZ and HC groups in the validation set.

Figure S1: Score plots of PCA in samples of QC, SZ, and HC groups.

Figure S2: Scatter box plots for total conjugated BAs, total primary BAs, and total secondary BAs in the discovery set.

Figure S3: Scatter box plots for total BAs and total unconjugated BAs in the validation set.

**Table S1** Abbreviations and taxonomic relations of bile acid variables.

| **Abbreviation** | **Description** |
| --- | --- |
| TBAs | Concentration of total bile acids, the sum of all the 40 BAs measured. |
| Total primary BAs | Concentration of all primary BAs, including CA, CDCA, GCA, GCDCA, TCA, and TCDCA. |
| Total secondary BAs | Concentration of all secondary BAs, including 12-ketoLCA, 3-DHCA, 6-ketoLCA, 7-ketoDCA, 7-ketoLCA, alloLCA, apoCA, CDCA-3Gln, DCA, GDCA, GHCA, GLCA, GLCA-3S, GUDCA, HCA, HDCA, isoLCA, LCA, LCA-3S, NorCA, NorDCA, TDCA, THCA, TLCA, TUDCA, TαMCA, UCA, UDCA, βCA, βCDCA, βDCA, βMCA, βUCA, and βUDCA. |
| Total conjugated BAs | Concentration of all conjugated BAs, including GCA, GCDCA, TCA, TCDCA, GDCA, GHCA, GLCA, GLCA-3S, GUDCA, TDCA, THCA, TLCA, TUDCA, and TαMCA. |
| Total unconjugated BAs | Concentration of all unconjugated BAs, including CA, CDCA, 12-ketoLCA, 3-DHCA, 6-ketoLCA, 7-ketoDCA, 7-ketoLCA, alloLCA, apoCA, CDCA-3Gln, DCA, HCA, HDCA, isoLCA, LCA, LCA-3S, NorCA, NorDCA, UCA, UDCA, βCA, βCDCA, βDCA, βMCA, βUCA, and βUDCA. |
| G-conjugated BAs | Concentration of all glycine conjugated BAs, including GCA, GCDCA, GDCA, GHCA, GLCA, GLCA-3S, and GUDCA. |
| T-conjugated BAs | Concentration of all taurine conjugated BAs, including TCA, TCDCA, TDCA, THCA, TLCA, TUDCA, and TαMCA. |

**Table S2** Median (IQR) serum concentrations (nM) of 40 BAs in SZ and HC groups in the validation set.

| BAs | SZ  (n=49) | HC  (n=48) | ratio^a^ | *p*-value^b^ | *q*-value^c^ | VIP^d^ |
| --- | --- | --- | --- | --- | --- | --- |
| CA | 11.19 (24.64) | 23.27 (38.54) | 0.48 | 1.51E-02 | 4.32E-02 | 0.77 |
| GCA | 19.52 (13.45) | 36.14 (33.77) | 0.54 | 3.38E-03 | 1.50E-02 | 1.05 |
| TCA | 1.73 (2.38) | 3.55 (6.09) | 0.49 | 7.03E-03 | 2.56E-02 | 1.11 |
| **CDCA** | 15.41 (46.03) | 95.46 (122.48) | 0.16 | 6.64E-07 | 1.33E-05 | 1.96 |
| GCDCA | 123.65 (113.59) | 205.86 (406.99) | 0.60 | 3.67E-04 | 3.67E-03 | 1.49 |
| TCDCA | 11.82 (15.54) | 17.46 (18.02) | 0.68 | 2.71E-02 | 7.22E-02 | 0.71 |
| DCA | 44.39 (59.9) | 66.56 (77.33) | 0.67 | 7.52E-02 | 1.28E-01 | 1.02 |
| GDCA | 29.66 (49.98) | 53.83 (76.11) | 0.55 | 7.20E-02 | 1.28E-01 | 0.69 |
| TDCA | 3.24 (4.78) | 5.22 (7.7) | 0.62 | 3.23E-02 | 7.65E-02 | 0.65 |
| **UDCA** | 6.64 (13.32) | 18.01 (30.56) | 0.37 | 8.22E-04 | 6.58E-03 | 1.68 |
| GUDCA | 26.67 (43.07) | 45.83 (63.29) | 0.58 | 5.74E-02 | 1.15E-01 | 0.84 |
| TUDCA | 0.82 (0.81) | 1.02 (1.53) | 0.80 | 6.21E-02 | 1.18E-01 | 0.61 |
| LCA | 1.83 (2.5) | 2.89 (3.27) | 0.63 | 3.25E-02 | 7.65E-02 | 0.62 |
| GLCA | 1.88 (3.85) | 4.14 (6.85) | 0.45 | 1.28E-01 | 1.83E-01 | 0.39 |
| TLCA | 0.13 (0.47) | 0.15 (0.63) | 0.87 | 8.69E-01 | 8.69E-01 | 0.25 |
| HCA | 2.94 (4.02) | 4.87 (6.95) | 0.60 | 5.69E-03 | 2.28E-02 | 1.59 |
| GHCA | 2.56 (3.05) | 4.44 (4.36) | 0.58 | 3.75E-02 | 8.34E-02 | 0.70 |
| THCA | 1.64 (1.23) | 1.91 (1.34) | 0.86 | 7.67E-02 | 1.28E-01 | 0.43 |
| βUDCA | 29.66 (42.8) | 46.95 (51.41) | 0.63 | 8.96E-03 | 2.99E-02 | 1.07 |
| **βCDCA** | 11.63 (10.87) | 30.96 (33.25) | 0.38 | 2.79E-10 | 1.11E-08 | 2.11 |
| βDCA | 9.65 (15.56) | 14.51 (16.18) | 0.67 | 9.22E-02 | 1.42E-01 | 0.60 |
| βUCA | 0.22 (0.34) | 0.14 (0.24) | 1.57 | 1.28E-01 | 1.83E-01 | 0.56 |
| βCA | 0.93 (0.61) | 1.25 (1.13) | 0.74 | 1.51E-03 | 1.01E-02 | 1.17 |
| βMCA | 0.34 (0.61) | 0.32 (0.66) | 1.06 | 6.31E-01 | 6.47E-01 | 0.10 |
| UCA | 0.3 (0.34) | 0.19 (0.25) | 1.58 | 8.81E-02 | 1.41E-01 | 0.36 |
| TαMCA | 0.98 (1.01) | 1.69 (2.51) | 0.58 | 2.97E-03 | 1.48E-02 | 1.16 |
| alloLCA | 0.3 (0.45) | 0.35 (0.47) | 0.86 | 3.88E-01 | 4.72E-01 | 0.18 |
| isoLCA | 1.75 (3.28) | 4.87 (8.94) | 0.36 | 2.43E-03 | 1.39E-02 | 1.00 |
| NorDCA | 0.63 (0.87) | 0.82 (0.98) | 0.77 | 3.27E-01 | 4.22E-01 | 0.31 |
| 6-ketoLCA | 0.64 (0.49) | 0.74 (0.5) | 0.86 | 1.45E-01 | 2.00E-01 | 0.68 |
| **7-ketoLCA** | 1.26 (2.36) | 3.86 (6.65) | 0.33 | 3.64E-04 | 3.67E-03 | 1.88 |
| 12-ketoLCA | 1.76 (2.5) | 2.45 (3.59) | 0.72 | 2.30E-01 | 3.06E-01 | 0.97 |
| apoCA | 2 (6.7) | 1.72 (2.19) | 1.16 | 5.53E-01 | 5.82E-01 | 0.98 |
| LCA-3S | 2.29 (3.66) | 2.62 (6.61) | 0.87 | 4.35E-01 | 4.83E-01 | 0.86 |
| GLCA-3S | 56.01 (98.56) | 60.87 (89) | 0.92 | 4.33E-01 | 4.83E-01 | 0.69 |
| HDCA | 1 (0.62) | 1.16 (0.7) | 0.86 | 4.02E-01 | 4.73E-01 | 0.10 |
| NorCA | 2.02 (2.26) | 1.43 (1.44) | 1.41 | 4.50E-02 | 9.48E-02 | 0.89 |
| **3-DHCA** | 0.24 (0.3) | 0.37 (0.72) | 0.65 | 1.35E-02 | 4.15E-02 | 1.48 |
| 7-ketoDCA | 0.82 (1.13) | 0.7 (0.76) | 1.17 | 3.89E-01 | 4.72E-01 | 0.10 |
| CDCA-3Gln | 3.88 (5.83) | 4.16 (4.94) | 0.93 | 5.06E-01 | 5.47E-01 | 0.74 |

Abbreviations: BAs, bile acids; VIP, variable importance in the projection; CA, cholic acid; GCA, glycocholic acid; TCA, taurocholic acid; CDCA, chenodeoxycholic acid; GCDCA, glycochenodeoxycholic acid; TCDCA, taurochenodeoxycholic acid; DCA, deoxycholic acid; GDCA, glycodeoxycholic acid; TDCA, taurodeoxycholic acid; UDCA, ursodeoxycholic acid; GUDCA, glycoursodeoxycholic acid; TUDCA, tauroursodeoxycholic acid; LCA, lithocholic acid; GLCA, glycolithocholic acid; TLCA, taurolithocholic acid; HCA, hyocholic acid; GHCA, glycohyocholic acid; THCA, taurohyocholic acid; βUDCA, 3β-ursodeoxycholic acid; βCDCA, 3β-chenodeoxycholic acid; βDCA, 3β-deoxycholic acid; βUCA, β-ursocholic acid; βCA, 3β-cholic acid; βMCA, β-muricholic acid; UCA, ursocholic acid; TαMCA, tauro α-muricholic acid; alloLCA, allolithocholic acid; isoLCA, isolithocholic acid; NorDCA, 23-nordeoxycholic acid; 6-ketoLCA, 6-ketolithocholic acid; 7-ketoLCA, 7-ketolithocholic acid; 12-ketoLCA, 12-ketolithocholic acid; apoCA, apocholic acid; LCA-3S, lithocholic acid-3-sulfate; GLCA-3S, glycolithocholic acid-3-sulfate; HDCA, α-hyodeoxycholic acid ; NorCA, norcholic acid; 3-DHCA, 3-dehydrocholic acid; 7-ketoDCA, 7-ketodeoxycholic acid; CDCA-3Gln, chenodeoxycholic acid-3-β-d-glucuronide. ^a^Ratios were calculated from the intra-group medians of BAs between SZ and HC groups. ^b^*P*-values were calculated by Mann−Whitney *U* tests. ^c^FDR adjusted *q*-values were calculated based on *p*-values estimated by Mann−Whitney *U* tests. The bold BAs indicate the 5 validated differential BAs in the discovery set.


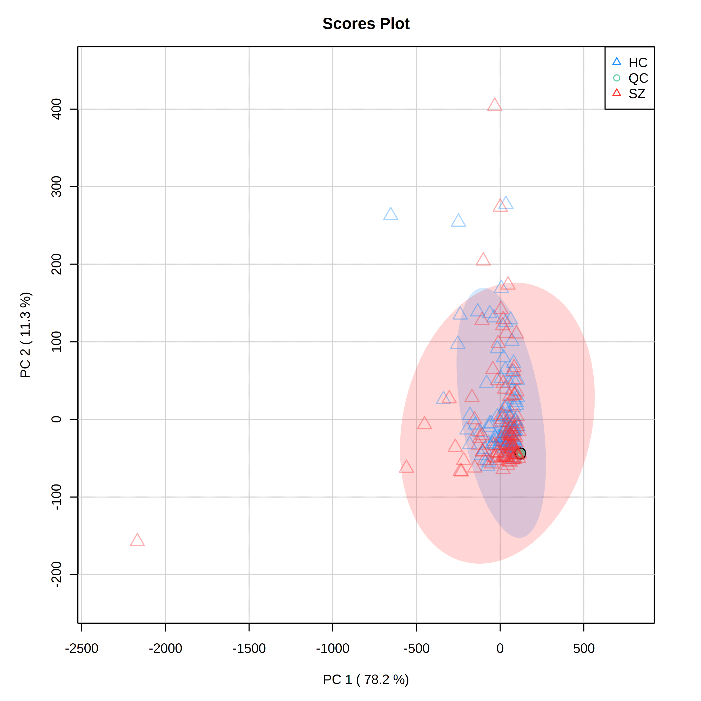


**Figure S1.** Score plots of PCA in samples of QC, SZ and HC groups, with 95% confidence ellipses drawn.

**
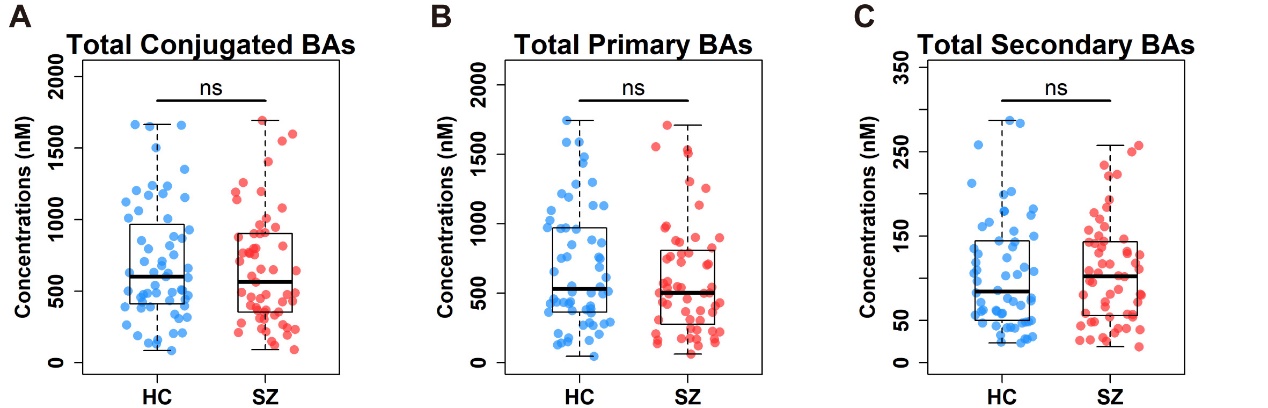
**

**Figure S2.** Scatter box plots for total conjugated BAs (A), total primary BAs (B), and total secondary BAs (C) in the discovery set. HC, healthy controls; SZ, schizophrenia; ns, not significant. The comparisons between the two groups were conducted by the Mann–Whitney *U* tests, excluding outliers. Center lines of box plots show median values, box hinges indicate 1st and 3rd quartiles, and whisker represent the furthest data points within 1.5 interquartile ranges of the hinges.


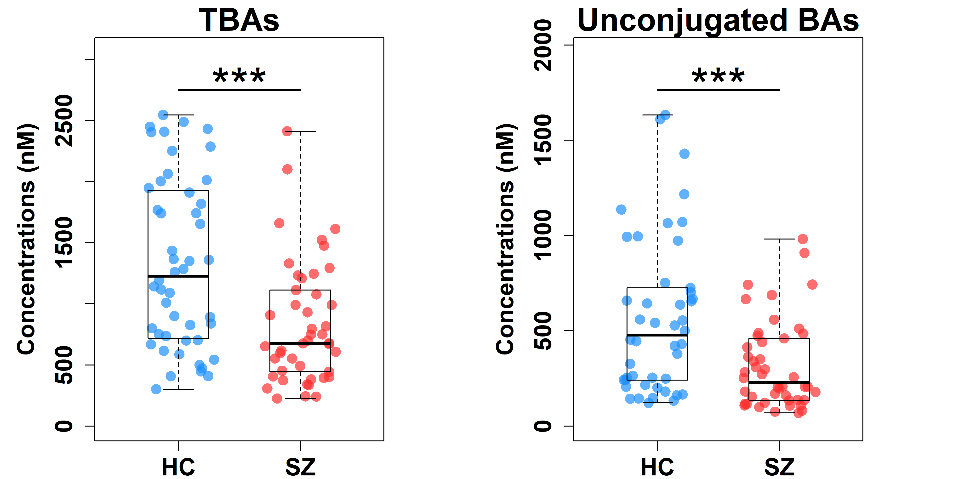


**Figure S3.** Scatter box plots for total BAs (TBAs) and total unconjugated BAs in the validation set. The comparisons between the two groups were conducted by the Mann–Whitney *U* tests, excluding outliers. *** *p* < 0.001. Center lines of box plots show median values, box hinges indicate 1st and 3rd quartiles, and whisker represent the furthest data points within 1.5 interquartile ranges of the hinges.
